# Removal of Parotid Tumor

**Published:** 1861-05

**Authors:** R. L. Rea

**Affiliations:** Prof. Anat., Rush Medical College


### REMOVAL OF PAROTID TUMOR.

BY R. L. REA, M. D.,

*Prof. Anat., Rush Medical College.*

Mr. J. B., of Earlville, Ill., applied to me on Dec. 6 last on account of a tumor in his left parotid region. He was a young man, 22 years of age, farmer by occupation, of robust appearance, and had always enjoyed uninterrupted health. He received a blow on the seat of the present enlargement two years since. Soon after he noticed the beginning of the present tumor, which has continued to grow steadily and painlessly, but slowly, ever since, until within two months ago, when it began to increase much more rapidly, and, becoming alarmed, he desires its removal.

The tumor now occupies the space immediately below the ear, extending from it above, to a line parallel to and half an inch below the lower jaw, and from an inch behind the posterior margin of the sterno mastoid, forwards overlapping the ramus of the lower jaw to the same distance, making a tumor double the size of a hen's egg.

The tumor was nodulated, readily moveable, though it seemed to be attached by a small part of its deep surface, behind the ramus of the jaw. There was no tenderness on pressure, and while it seemed quite firm, there was undistinct fluctuation over a portion of it. It has never been painful in the slightest degree; no discoloration of the integuments, to which it is closely attached, but seems, on a careful examination, to be free on its under surface, with the exception before mentioned.

On the following day, assisted by Dr. Lynn, and Messrs. Comstock, Minesinger and Mehler, students of Rush Medical College, I proceeded to remove it.

I made a crucial incision over the body of the tumor, found it enclosed by an almost cartilaginous capsule; after opening which, I had no difficulty in enucleating it, the point of adhesion, which I correctly supposed to exist behind the ramus, being carefully separated by the point of my finger.

No difficulty was had in its removal, and no vessels divided requiring ligature. The wound was closed by suture, except the lower section, which was left open to allow free exit to pus.

Dec. 8—Swelling considerable; ordered cold water dressings.

Dec. 9—Swelling still increasing; headache, with some fever, and wound quite painful. Upon examination I found the wound had healed almost entirely by first intention—the portion without suture healing with the balance, and the consequence was an accumulation of pus. A free discharge of matter followed opening it, removed the other stitches and and placed a tent in it. Ordered a saline cathartic and continuance of cold water.

At my visit the following day I found his febrile symptoms much abated; wound discharging; swelling diminishing, and from this no unusual symptoms occurred, and on the 12th, five days after the operation, he returned home.

The interest attaching to this case is in the relations of the tumor to the Parotid Gland.

It might readily have been mistaken for the Parotid from its situation and removed as such, while in reality it had no connection with it except at a very small part of its deep surface. The lower portion of the Parotid was found atrophied from pressure by the tumor, but that it was not enlargement of the gland itself, was abundantly proven in the fact that none of the necessary results followed its removal.

It will be recollected that the formation of the External Jugular vein, by the union of the Temporal, Maxillary and Posterior Auricular veins takes place in the substance of this gland.

The Auricularis Magnus nerve, the largest ascending branch of the superficial cervical plexus, breaks up into its distributive branches, many of which join the Facial nerve, upon the Parotid.

The Auriculo-temporal, a branch of the fifth pair of nerves, passes behind the neck of the jaw upwards to the side of the head under cover of the Parotid. Whilst opposite the neck of the lower jaw it sends branches to join the Facial. The Internal Carotid artery and Internal Jugular vein rest closely against its inner surface.

The External Carotid artery passes upward directly through the gland, and while imbedded in its substance breaks up into its terminal branches, Internal Maxillary and Temporal, and gives off its Transverse Facial branch.

The Parotid completely embraces the artery and extends some distance beyond it, lying between the neck of the lower jaw and Mastoid process, occupying the posterior portion of the Glenoid cavity and resting against the Styloid process and Styloid muscles. It also embraces the ramus of the lower jaw extending beneath it forward between the Pterygoid muscles.

But by far the most serious complication in the relations of the Parotid, in reference to its extirpation, is the Facial division of the seventh pair of nerves. This nerve is one of motion, supplying the muscles of the side of the face. It escapes from the cranium at the Stylo-mastoid foramen, then passing forwards, divides behind the ramus of the jaw in the substance of the Parotid gland into its terminal and distributive filaments, which, spreading out in it, radiate to all parts of the side of the face, extending their supply from above the eyebrow to below the chin.

It will be seen by examining the relations here mentioned that the removal of the Parotid, while it is not impossible by any means, is nevertheless one of the most important and delicate in Surgery. The Facial nerve, from the delicacy of its filaments, and the intimate manner in which they are blended with the structure of the gland, renders their preservation in the removal of the entire Parotid an *impossibility*.

The complete removal of the Parotid will invariably be followed by paralysis of the muscles supplied by the Facial nerve of the affected side, the measure of paralysis being determined by the amount of gland removed, and consequent destruction of nervous substance. Any one who has carefully examined the relations of the gland on the dead subject, must have been struck with the amount of care required to detect any fair proportion of the distribution filaments of the nerve—much less to preserve them.

How vastly more difficult must be the task when the parts are filled with blood, bleeding at every point, and especially in that wedge-shaped mass of it filling the sulcus between the jaw and mastoid process, reaching behind the ramus, directly through which pass the primary trunks of this most important nerve. Add to this the fact that the diseased action necessitating its removal, has most probably caused adhesion to surrounding parts, and we may form some idea of the difficulties in the way of its removal leaving the nerve in its entirety.

These remarks and objections are intended of course to apply to normal formations and distribution of the several parts. I could imagine a case in which we had an abnormally small and favorably placed Parotid, the Facial nerve for instance passing entirely beneath it, when the termination might be entirely favorable. I think the cases where it is reported to have been removed, paralysis not following, must have been thus abnormally favorable.

I have said nothing of the External Carotid, the wounding of which is a matter of comparatively little consequence.

